# Validating the Alberta Context Tool in a multi-site Australian Emergency Department nurse population

**DOI:** 10.1371/journal.pone.0215153

**Published:** 2019-04-09

**Authors:** Verena Schadewaldt, Benjamin McElduff, Catherine D’Este, Elizabeth McInnes, Simeon Dale, Anoja Gunaratne, Janet Squires, Dominique A. Cadilhac, Sandy Middleton

**Affiliations:** 1 Nursing Research Institute, Australian Catholic University and St Vincent’s Health Australia Sydney, Darlinghurst, New South Wales, Australia; 2 National Centre for Epidemiology and Population Health (NCEPH), Australian National University, Canberra, Australian Capital Territory, Australia; 3 School of Medicine and Public Health, Faculty of Health and Medicine, The University of Newcastle, Callaghan, New South Wales, Australia; 4 School of Nursing, University of Ottawa, Ottawa, Ontario, Canada; 5 Clinical Epidemiology, Ottawa Hospital Research Institute, Ottawa, Ontario, Canada; 6 Stroke and Ageing Research, School of Clinical Sciences at Monash Health, Monash University, Clayton, Victoria, Australia; 7 The Florey Institute of Neuroscience and Mental Health, University of Melbourne, Parkville, Victoria, Australia; Victoria University, AUSTRALIA

## Abstract

The organisational context of healthcare settings has an essential role in how research evidence is used in clinical practice. The Alberta Context Tool (ACT) measures 10 concepts of organisational context with higher scores indicating a more positive work environment and potentially better use of research evidence in patient care. We assessed the psychometric properties of the ACT in Emergency Departments (EDs). This validation study was conducted as part of a multi-centre trial of triage, treatment and transfer (T^3^ Trial) of patients with stroke admitted to EDs. Stratified sampling with proportional allocation was used to recruit ED nurses from 26 participating hospitals at baseline. Nurses completed a survey containing the ACT. Structural validity was investigated by exploratory and confirmatory factor analysis. Reliability was assessed using Cronbach’s alpha and intraclass correlation coefficients. Item-rest correlations and the average inter-item correlations were also assessed. 558 ED nurses completed the survey, comprised of 433 surveys without missing data. Our exploratory factor analysis produced a 14-factor structure, explaining 62% of variance of organisational context. For eight of ten concepts, item loadings matched the factor structure of the original ACT. Confirmatory factor analysis of the 10 ACT concepts showed moderate model fit (p = 0.001, root mean square error of approximation: 0.049, standardised root mean squared residual: 0.048). Cronbach’s alphas showed very good internal consistency for nine of ten ACT concepts (α>0.7; 0.45–0.90). Item-rest correlations indicated that most ACT items (50 of 56 items) within any concept related well to the total score of the concept. Average inter-item correlations indicated potential redundant items for three concepts (feedback processes, leadership, staffing) that were above the threshold of 0.5. While identifying a few shortcomings for some ACT concepts in an ED context, the majority of findings confirm reliability and validity of the original ACT in an Australian population of ED nurses.

## Introduction

Implementing research findings into clinical practice is crucial to provide high quality care. However, researchers and clinicians struggle to achieve and sustain the translational process from evidence into practice despite available implementation strategies [1–3]. Implementation research has identified context as influential in terms of health professionals’ research utilisation [4–7] and the implementation and dissemination process of research-based knowledge in a clinical setting [8]. ‘Context’ refers to a number of human factors such as leadership, staff interaction and staffing levels and also structural factors such as workplace culture, available resources and physical work space [9]. In this paper we refer to context as ‘organisational context’ of a health care setting comprising all of the above factors. Evidence is growing that supports that the use of research to inform practice is greatest in those settings with positive perceptions by staff of their workplace context [10–12]. Conducting an assessment of organisational context can identify those factors that may impact upon whether or not evidence-based resources such as clinical practice guidelines will be used by clinical staff [11]. Staff are then enabled to address the modifiable factors of organisational context, thus helping to provide the optimum environment for the introduction of evidence based practice [13].

The Alberta Context Tool (ACT) was developed to assess the organisational context of healthcare settings and research utilisation behaviour of health professionals [14]. It measures those aspects of the organisational context that have an impact on how well clinicians may use research in clinical practice [14]. The ACT has been broadly used in research projects to assess organisational context in acute and long-term care facilities as follows: 1) to identify and confirm associations between context and research use or adherence to best practice by clinical staff, which showed that a positive organisational context fosters research use [10–12, 15–18]; 2) to identify associations between context and patient outcomes, which showed improved health outcomes for nursing home residents in facilities with higher context scores [19]; 3) to identify associations between context and health care delivery, which showed staff in nursing homes with lower context scores reported more often being under pressure when delivering care [20]; and 4) to identify associations between context and staff characteristics, which showed that there was no relationship between organisational context and years of clinical practice [21, 22].

To our knowledge, there is no research that has reported the application of the ACT by health professionals working in the Emergency Department (ED). One study protocol has been published, stating the planned application of the ACT in paediatric EDs but results have not been published as of January 2018 [23]. The majority of studies have been undertaken in nursing homes and acute paediatric wards that differ from the high acuity work environment of an ED. ED staff are under constant pressure to make quick decisions, address a multitude of diseases and injuries whilst working with various disciplines [24, 25]. Limited research using the ACT has been undertaken in Australia, including its application in general medical wards [22] and stroke units [26, 27]. Therefore, we undertook a validation study of the ACT in an ED nurse population to determine the psychometric properties of the ACT within this setting and clinician group.

## Methods

### Design and setting

A cross-sectional survey was used for this validation study that was part of a larger implementation project, the T^3^ Trial, a cluster randomised controlled trial undertaken to evaluate the impact of a multidisciplinary intervention to improve treatment, triage and transfer of patients with stroke in the ED on 90-day patient outcomes [28]. The study was undertaken in 26 hospitals across three Australian states and one territory. The survey was undertaken prior to commencement of any pre-implementation data collection, as part of a process evaluation to help explain how the organisational context may have contributed to the subsequent success or failure of uptake of the T^3^ intervention.

### Ethics approval

Approval from the Human Ethics Research Committees from the following sites have been obtained: Australian Catholic University (2012 16N), Sydney Local Health (Royal Prince Alfred Hospital Zone) (HREC/12/RPAH/32) (NSW Lead Ethics Committee); ACT Government Health Directorate Human Research Ethics Committee (HREC) (ETH.1.13.009); Uniting Care Health HREC (1231); The Alfred, Melbourne HREC (503/12); Northeast Health Wangaratta HREC; and South West Healthcare Warrnambool HREC (5/2012).

### Recruitment, sample and data collection

Nurses on the ED staff roster at participating hospitals were eligible to participate, including night shift staff. Casual staff and agency staff were excluded. The nurse unit manager (NUM) of each ED was contacted initially to identify the number of nurses working in their ED, number of full-time and part-time nurses and number of enrolled and registered nurses. Based on the total number of nursing staff, EDs were classified as small (≤ 50 nurses) or large (> 50 nurses). At small EDs, 40% of nurses were sampled, resulting in a projected sample size of approximately 80 nurses from five small EDs. At larger EDs, 20% of the nurses were sampled, resulting in approximately 500 nurse participants with a total projected sample of approximately 580 nurses. We used stratified random sampling with proportional allocation to obtain a sample representative of the number of nurses qualified as enrolled nurses (EN) or registered nurses (RN), and working full-time or part-time within each ED.

Each NUM was provided with a set of surveys, a set of replacement surveys, an administration protocol, and a list which specified the total number of surveys required from the hospital and how many surveys were to be distributed to each type of nurse (EN/RN; full-time/part-time). The NUM or another delegated ED staff member was responsible for distribution of the paper-based self-administered survey. They self-selected eligible staff within each nurse category. The administration protocol stated that if a staff member declined to fill out a survey, the person distributing the surveys was to give the survey to a nurse with the same category (e.g. RN full-time replaced with RN full-time). Furthermore, if a survey was not returned within three weeks, a replacement survey was to be provided to another nurse within the relevant category.

An information sheet explained the purpose of the study and the survey. Surveys could be returned to the site contact in a sealed envelope or returned directly via reply-paid envelope to the T^3^ research office. The return of the survey implied consent to participate. The site contact gave verbal reminders to complete the survey at seven and fourteen days following the survey distribution.

### Data collection instrument

The ACT nurse version [29] was used for this study following permission from the tool developers. The ACT was constructed originally with two foci in mind. First, in order to promote its use in clinical settings, the tool needed to be quick to complete [14]. Secondly, the tool had to assess modifiable elements of context in order to enhance the implementation and uptake of research evidence in practice [14]. The development of the ACT was based on the theoretical construct of context as outlined in the Promoting Action on Research Implementation in Health Services (PARIHS) framework [7] and other literature reporting contextual factors that play a role in research utilisation by health care providers. The PARHIS framework is a theoretical model to describe and assess how research evidence is implemented in practice and the PARHIS context domain comprises concepts of culture, leadership and evaluation [7]. These concepts were expanded by the tool developers to 10 concepts of organisational context: (1) leadership, (2) culture, (3) feedback processes (evaluation), (4) connections (social capital), (5) formal interactions, (6) informal interactions, (7) structural and electronic resources, (8) staffing, (9) time and (10) space, with these latter three sub-concepts of organisational slack [14, 30]. A number of items were developed to measure each concept. An exploratory factor analysis (EFA) during the tool development phase resulted in a 13-factor structure that explained 59.3% of variance of organisational context [14] with variances around 60% and above commonly accepted as satisfactory [31]. Eight of ten concepts are represented by a single factor, with the other two concepts comprising more than one factor (resources comprise three and informal interactions two factors). Following pilot-testing and refinement in samples of nurses and other health professionals, the final tool contained 56 items. Reliability testing resulted in Cronbach’s alpha at or above the conventional standard of 0.7 for 9 factors and showed acceptable values of > 0.54 and < 0.7 for 4 factors [14].

All ACT items are rated on five-point Likert scales. Response options for leadership, culture, feedback processes, connections, staffing and space items are *strongly disagree*, *disagree*, *neither agree nor disagree*, *agree*, *strongly agree* while items assessing formal and informal interactions, structural/electronic resources and time are classified as *never*, *rarely*, *occasionally*, *frequently and almost always*. Higher scores indicate a more positive perception of the work context. The ACT is not designed to produce a single overall score of context and the tool developers advise analysing each concept as an individual scale [32].

Our survey also obtained demographic information on age, gender, time since qualified, length of time working in ED, professional position, level of education, specialisation in emergency nursing or critical care nursing, employment status (full-time, part-time) and working rotating shifts.

### Statistical analysis

The statistical analysis was carried out with STATA 14 [33] and R statistical software [34]. In accordance with scoring instructions, the ACT items from three concepts (formal interactions, informal interactions, structural/electronic resources) were recoded to 0 for never/rarely, to 0.5 for occasionally and to 1 for frequently/almost always and an overall sum for each concept was calculated [32]. The remaining concepts kept the item scoring of 1 to 5 and a mean score was calculated for each concept. For ACT concepts, items with more than 10% of observations with missing data, or with more than 90% of observations in a single category were reviewed and considered as potentially unnecessary or irrelevant items in this population [35]. Sociodemographic characteristics of the sample are reported as number and percentages for categorical variables and means and standard deviations for continuous variables. Cases with missing data were excluded from the analysis.

### Structural validity

Since the ACT has not been used in a sample of ED nurses, we undertook an exploratory factor analysis to investigate the factor structure of the instrument without imposing any specific framework. Principal component analysis (PCA) was performed followed by varimax rotation. In order to follow the original ACT validation study, we set the eigenvalue cut-off score as ≥1.0 for factors to be retained in the model and factor loading coefficients of ≥ 0.35 for items to be retained in the factor [14].

A confirmatory factor analysis (CFA) was also performed to verify the ACT’s original structure in our ED nurse population. We modelled the data to the ACT factor structure within a structural equation modelling framework and used three measures to assess model fit: the root mean square error of approximation (RMSEA); the standardised root mean squared residual (SRMSR); and the comparative fit index (CFI). A RMSEA of < 0.07, an SRMSR < 0.8 and a CFI > 0.95 indicate good fit [36]. Additionally, we report the p-value of the model χ^2^ test, which tests for difference between the sample and fitted covariance matrices. This test is sensitive to sample sizes > 200 and thus likely to reject the null hypothesis, that the model fits exactly. Three of the ACT concepts (informal interactions, formal interactions and structural/electronic resources) were developed as a comprehensive list of potential interactions staff may have had, or available resources that they utilised. Rather than representing true scales with conceptually related items, the informal/formal interaction and resources concepts included lists of independent items assessing various ways of interaction between staff and use of different resources [15]. Concept scores for these three concepts were calculated as the sum or count of the number of items within each concept that respondents participated in or used. Since a factor analysis groups conceptually related items under a factor, it was unlikely that the factor analysis would show an acceptable model fit. ([15, 37]). In order to identify the impact of the ‘count-based’ or non-scaled concepts on the model fit, we tested three models with i) all 10 ACT concepts, ii) seven concepts based on true scales and iii) three concepts comprising the count-based concepts.

### Internal reliability

Cronbach’s alpha was used to assess internal consistency of the factors from the 10 concepts identified in the original study. Reliability measures and their cut off scores for good scale quality are summarised in Table 1. We also assessed the change in Cronbach’s alpha associated with removal of each item from the concept to determine if the reliability of the scale would improve by deleting individual scale items. Values of > 0.70 have been cited as acceptable, whereas values of > 0.80 are preferable [38, 39].

**Table 1:** Good psychometric properties for scale quality.

Reliability measures	Criteria for reliable measure
Cronbach’s alpha	≥ 0.7 [35,36]
Cronbach’s alpha if item deleted	Alpha is not decreased by deleting any item
Item-rest correlation (corrected item correlation)	≥ 0.3 [14]
Average Inter-item correlation coefficient	Between 0.15–0.5, preferred values between 0.4–0.5 [37]
Intraclass correlation coefficient	≥ 0.10 indicates strong perceptual agreement [15]

Internal consistency was also assessed using item-rest correlations, i.e. correlations between one scale item and the remaining items of the concept. It is assumed that items contributing to the total of the scale correlate positively with the overall scale. In reference to the original ACT development, a correlation coefficient of > 0.3 is considered acceptable for items to remain in the scale [14].

In addition, inter-item correlations were assessed, and coefficients between 0.15 and 0.5 were considered as acceptable [40]. Correlations below 0.15 may indicate that items do not relate well to the concept they are measuring and coefficients above 0.5 may indicate that items are redundant because they are measuring the same aspect of the concept [40]. For specific constructs such as the 10 concepts of organisational context, coefficients of 0.4–0.5 are preferable [40].

Since the ACT is completed by individual health professionals but is commonly used as a measure of organisational context of an entire unit (ED) in the analysis, it is important to establish that individual-level data from health professionals reflect unit-level context. The intraclass correlation coefficient (ICC) is an estimate of agreement of individuals’ scoring about the mean score within a unit [41]. One-way random effects ANOVA, defined as ICC (1,1) by Shrout and Fleiss (1979) was used to determine if responses from individuals could be aggregated to unit-level, i.e. if high interrater agreement exists within the respective EDs [41]. ICC (1,1) values range from 0 (no agreement) to 1 (perfect agreement), however, in applied research an ICC (1,1) ≥ 0.10 is indicative of perceptual agreement among individuals within the same unit [15]

## Results

### Sample characteristics

Overall 558 completed surveys were returned. Five EDs provided more than their specified quota of surveys, 14 provided fewer and seven provided the correct quota. The total number of surveys distributed, the number of nurses approached to complete a survey and the number of nurses who declined to complete a survey are unknown, therefore we are unable to provide a response rate for the study. The demographic characteristics of the sample are shown in Table 2. The majority of respondents (n = 413, 74%) were female with almost half of the participants aged between 25 and 34 years (n = 253, 45%). The mean number of years nurses worked in the ED was 6.1, ranging from 0.1–38 years. A third of the nurses were educated at Bachelor’s degree level (n = 186, 33%) with 39% of the nurses (n = 217) holding a postgraduate certificate or diploma degree. More than half (n = 325, 58%) of the nurses worked in the role of a registered nurse with other roles listed in Table 2.

**Table 2:** Nurse demographics.

ED Nurse characteristics		Number (%)* n = 558
**Gender**	Female	413 (74%)
	Male	78 (14%)
**Age groups**	≤ 24	57 (10%)
	25–34	253 (45%)
	35–44	94 (17%)
	45–54	70 (13%)
	≥ 55	28 (5%)
**Highest education**	Hospital-based certificate	15 (3%)
	Certificate III or IV (TAFE qualification)	12 (2%)
	Diploma (TAFE qualification)	17 (3%)
	Bachelor’s degree	186 (33%)
	Graduate Certificate	137 (25%)
	Graduate diploma	80 (14%)
	Master’s degree	48 (9%)
	PhD, DN	-
**Profession**	Enrolled nurse	4 (1%)
	Endorsed enrolled nurse	27 (5%)
	Registered nurse	325 (58%)
	Registered nurse clinical specialist	93 (17%)
	Clinical support nurse	3 (1%)
	Clinical nurse educator	16 (3%)
	Nurse practitioner	5 (1%)
	Nurse unit manager	6 (1%)
	Associate nurse unit manager	20 (4%)
**Years worked in trial ED [Mean, SD, Min-Max]**		6.1, 6.2, 0.1–38

*Numbers may not add up to total sample size due to missing values

Between 10% and 12% of observations had missing data for demographic information and 55 (9.9%) nurses did not complete any information on the demographic part of the survey. For the ACT, there were 433 nurse surveys with no missing data. Missing data for individual ACT items was minimal, ranging from 4 (0.7%) to 20 (3.6%). Response patterns showed no single category with more than 90% of observations.

### Structural validity—Factor analyses

Our exploratory factor analysis produced a 14-factor structure, explaining 62% of variance associated with organisational context (Table 3). S1 Appendix highlights those loadings (blue cells) that match the original item loadings. With the exception of formal interactions and informal interactions, the items of the remaining 8 ACT concepts loaded onto their corresponding factor (S1 Appendix).

**Table 3:** Exploratory factor analysis, eigenvalues and proportion of variance.

Factor	Eigenvalue^	Proportion of variance (%)	Cumulative variance (%)	No of items	Factor Loadings (Min-Max)
**1**	4.38	7.82	7.82	6	0.74–0.81
**2**	4.05	7.23	15.05	7	0.36–0.83
**3**	3.16	5.65	20.70	6	0.55–0.70
**4**	2.94	5.25	25.95	6	0.53–0.65
**5**	2.52	4.50	30.45	5	0.39–0.74
**6**	2.37	4.24	34.69	4	0.37–0.80
**7**	2.33	4.16	38.85	4	0.54–0.72
**8**	2.27	4.05	42.90	4	0.46–0.71
**9**	2.15	3.84	46.74	4	0.51–0.71
**10**	1.99	3.56	50.30	3	0.38–0.85
**11**	1.99	3.56	53.86	3	0.51–0.79
**12**	1.99	3.55	57.41	3	0.38–0.90
**13**	1.35	2.41	59.81	1	0.79
**14**	1.26	2.24	62.06	2	0.38–0.78

^By convention, eigenvalue here refers to the sum of squared loadings for each rotated factor, which is not an eigenvalue of the rotated loading matrix in a technical sense

#### Factor loadings

For the first four factors all items loaded on their corresponding concepts, which are leadership, feedback processes, connections and culture (S1 Appendix). Factor 4 comprises all items of culture with one item (*being a member of a supportive work group*) cross-loading onto leadership. However, this item has a low factor loading of 0.36 for the leadership factor. The combined variance for the first four factors explaining organisational context is 26% (Table 3). All items of the three organisational slack concepts–time, staffing, space–loaded on their relevant factors. One item of the concept space (*adequate space to provide patient care*) cross-loaded onto staffing with a factor loading of 0.51.

The four items of formal interactions loaded onto three factors with one item (*participation in team meetings*) not loading onto any of the retained factors at a loading of ≥ 0.35. Informal interactions were represented in three factors, one reflecting interactions with other clinicians, one reflecting interactions with professionals involved in research and one comprising two items, one of which (*occurrence of informal bedside teaching*) cross-loaded onto structural resources. This two-item factor was not part of the original ACT structure. Structural and electronic resources were represented in three factors, reflecting three sub-types of resources: 1) formal resources–*noticeboards*, *policies and clinical practice guidelines; 2)* external and electronic resources–*workshops*, *computerised decision support*, *email reminders and websites;* and 3) traditional resources–*library*, *textbook and journals*.

#### Confirmatory factor analysis results

Model fit statistics from the confirmatory factor analysis are presented for all three models in Table 4. The low p-values for all three models (p<0.001) indicate that the data do not fit the predefined models exactly. The RMSEA and SRMSR show acceptable fit for model one with all 10 ACT concepts and model two with the seven true scale concepts. Model three based on the three count-based concepts shows unacceptable fit with a RMSEA of 0.087 and a borderline acceptable fit with a SRMSR of 0.078. The CFI for all three models lies below the conservative cutoff of >0.95, however, model two is close to good model fit with a CFI of 0.924.

**Table 4:** Model fit statistics.

Model	n	p-value^#^	RMSEA	SRMSR	CFI
1, comprises all concepts	433	< 0.001	0.049	0.048	0.83
2, comprises seven true scale concepts	471	< 0.001	0.048	0.053	0.924
3, comprises three count-based concepts	487	< 0.001	0.087	0.078	0.659

^**#**^ p-value from model χ^2^ test; RMSEA: root mean square error of approximation; SRMSR: standardized root mean squared residual; CFI: comparative fit index.

### Reliability

#### Internal reliability

Cronbach’s Alphas for 9 of 10 ACT concepts were above 0.7, showing very good internal consistency (Table 5). An exception was the concept titled formal interactions that had an alpha of 0.45, which indicates weak internal consistency.

**Table 5:** Reliability measures (n = 433).

ACT Concept	No. Items	α	α when item deleted (min—max)	Item-rest correlations (min—max)	Average Inter-item correlation	ICC
Feedback	6	0.90	0.87–0.89	0.65–0.77	0.59	0.17
Leadership	6	0.88	0.84–0.87	0.59–0.75	0.54	0.18
Connections	6	0.80	0.76–0.79	0.51–0.64	0.41	0.01
Culture	6	0.79	0.75–0.77	0.50–0.60	0.39	0.08
Structural and electronic resources	10	0.75	0.71–0.74	0.27–0.48	0.23	0.09
Informal interactions	9	0.76	0.72–0.76	0.23–0.52	0.26	0.09
Time	4	0.74	0.63–0.71	0.49–0.63	0.42	0.16
Formal interactions	4	0.45	0.34–0.43	0.20–0.29	0.17	0.13
Staffing	2	0.88	0.78–0.78	0.78–0.78	0.78	0.31
Space	3	0.75	0.44–0.87	0.38–0.77	0.50	0.10

α: Cronbach’s alpha, ICC: Intraclass correlation coefficient

#### Item-total statistics

Cronbach’s Alpha did not increase if a particular item within the scale was deleted for 9 of 10 concepts, hence none of the items seemed redundant. The exception was for the organisational slack concept space. Cronbach’s alpha of 0.75 would increase to 0.87 if the item titled *we have adequate space to provide patient care* was deleted.

#### Item-rest correlations

In accordance with the original ACT validation, we chose an item-rest correlation coefficient of > 0.3 as indication for a reliable measure. Item-rest correlations indicate that most items (50 of 56 items) within a concept related well to the total score of the concept. Seven of the 10 ACT concepts had item-rest correlation coefficients > 0.3 for all items with the majority of items having coefficients of > 0.4 (Table 5). The concepts named feedback, leadership and staffing indicated the highest item-concept correlations with maximum coefficients ranging from 0.59 (leadership: *leader focuses on success*) to 0.78 (staffing: both items).

Six of 56 items had item-rest correlations below the cut-off score of 0.3. Informal interactions (*occurrence of hallway talk*) and structural/electronic resources (*library*) had one item each with values of 0.23 and 0.27, respectively. The concept of formal interactions had all four items scoring below the cut-off score (0.20–0.29), indicating weak correlations between the concept formal interactions and its items.

#### Inter-item correlations

The average inter-item correlation or item-to-item correlation ranged from 0.17 (formal interactions) - 0.78 (staffing) (see Table 5). Only three concepts (connections, time and space) lie within the preferred range of 0.4–0.5. One concept (culture) is just below the threshold of 0.4 whereas three concepts (leadership, feedback processes and staffing) lie above the cut-off coefficient of 0.5 for good scale measurement, indicating that items within these concepts measure the concept in very similar ways and could potentially be redundant. None of the concepts had average inter-item correlations under the cut-off of 0.15, indicating that all items related well to the concept.

#### Intraclass correlation coefficient

The ICC (1,1) shows some degree of agreement among nurses for all domains with strong agreement (ICC (1,1) ≥ 0.10) for six out of ten domains (Table 5). Therefore, nurse responses within one ED were very similar and can be aggregated to unit-level responses to reflect ED organisational context.

## Discussion

This validation study in a large sample of 558 ED nurses from 26 Australian hospitals confirms the structural validity and reliability of the ACT to assess organisational context within this setting. That means, the ACT can be applied in an ED setting to evaluate the structural and human factors that may impact on the use of research evidence by nurses. Reliability as well as validity measures met the standards for good scale quality for almost all items within the scale. We achieved a high number of returned surveys, further adding to the strength of these findings.

Compared to the paediatric nurse sample (n = 752) of the original study testing the ACT [14], our sample of ED nurses included more males (4% vs 14%), and nurses who had been working for fewer years on the trial unit (7.7 vs 6.1). Our sample also included more nurses with a Master’s degree (1.7% vs 9%). The original factor analysis was based on a sample of 704 paediatric nurses (after deletion of incomplete cases) from six hospitals [14] whereas our sample (with a complete dataset) consisted of 433 nurses from 26 EDs making it one of the largest hospital samples in which the ACT has been used and thus contributing to context assessment of emergency care settings. The results of our validation study corroborate the application of the ACT in a population of ED nurses, which to our knowledge has not been previously investigated.

### Validity and reliability

Our exploratory factor analysis produced a 14-factor solution that accounted for a total of 62% of the variance associated with organisational context. For 8 of 10 concepts, item loadings matched the original factor structure. This finding mirrors well the scale structure of the ACT. The first report about the ACT described an exploratory factor analysis resulting in 14 factors based on 51 items and explaining 70% of variance of organisational context [30]. After refinement of the ACT, a later pilot study reported a 13-factor structure based on 56 items and accounting for 59% of variance [14].

In regards to internal reliability, alpha coefficients in our study were at or above the standard of 0.7 for 9 of 10 concepts. Similarly, previous validation studies found Cronbach’s alphas ≥ 0.7 for 7 of 8 concepts (organisational slack comprising time, staff and space) [30], for 9 of 13 concepts (based on the 13-factor structure) [14] and for 8 of 10 concepts in a sample of healthcare aides in nursing homes [15]. Two studies using translated versions of the ACT in non-Canadian populations presented Cronbach’s alphas of ≥ 0.7 for 10 of 13 concepts (based on the 13-factor structure) in a German population of health professionals in nursing homes [42] and for 5 of 8 concepts (organisational slack comprising time, staff and space) in a Swedish population of nursing home nurses [43]. In line with these studies, the ICCs for the ACT concepts showed high agreement among ED nurses within their respective ED, supporting the ACT’s purpose to assess the nurses’ specific organisational context. Most recently, Squires et al. re-analysed the ACT data from five different datasets, collected from their own research in nursing homes and paediatric hospitals but also from other researchers using the tool in acute adult hospitals and community care [37]. These studies further confirm internal reliability of the ACT.

Our findings align well with results from initial ACT validation studies in differing professional groups. In particular, for the concepts feedback processes, leadership, connections, staffing and time, all items load exclusively on one factor, confirming the item structure for these concepts. Apart from one item that is cross-loading from culture at a negligible low loading of 0.38, the six culture items also load onto one factor. The concepts culture, leadership and feedback processes have been key concepts in implementation research since they were used to define context in the PARIHS framework [7]. All of these concepts show a high degree of internal consistency with reliable alpha measures (apart from space) and item-rest correlations of > 0.3, meaning that all items relate well to the overall concept. However, limitations for internal reliability were seen for leadership, feedback processes and staffing with average inter-item correlations above 0.5 that indicate that items within these concepts measure the constructs in very similar ways and thus may capture a rather narrow focus of the construct. Potentially, in an ACT ED nurse version, some of these items could be removed, as they may not add much to the measurement of these concepts.

When considering the three sub-concepts time, space and staff combined as organisational slack, all items match the original item-structure of organisational slack with one item from space (*adequate space to provide patient care*) cross-loading onto staff. The cross-loading of the space item is not surprising as it fits the staff items on the factor that measures sufficient means (space and staff) to provide patient care. Interestingly, as shown by the reliability assessment, by deleting the *adequate space to provide patient care* item Cronbach’s alpha for space would increase. This item also has a low item-rest correlation of 0.38, indicating a weak correlation of this item to the overall scale. These findings are in accordance with other ACT validation studies that showed low alpha coefficients [15, 44] and low loadings [44] for the *adequate space* item. However, it appears appropriate to keep this item in the scale as it is conceptually essential to assess the organisational context regarding space. We speculate that adequate space in the ED may be difficult to capture with the ACT items because EDs have different spatial allocations for resuscitation, assessment or fast track patients and nurses move across various bays within the ED.

Three ACT concepts (resources, formal and informal interactions) were developed to rate the frequency of use of resources or frequency of interactions and their mean concept scores are derived from a sum or count-based method, which is different from those of the other concepts. Their scales represent lists with potentially independent items within one concept rather than representing true scales with conceptually related items [37]. Since a factor analysis examines relationships between items, it is reasonable to expect that items of these concepts do not load on one factor and that items have low item-rest correlations, indicating that items measuring this concept do not relate strongly with each other.

This was particularly evident for formal interactions in our study with three items loading onto three separate factors and one item (*participation in team meetings*) not loading onto any factor at a coefficient of > 0.35. The item *attendance at continuing education outside the hospital* loaded onto factor 5 that represented traditional resources such as *library*, *textbook*, *journals*. It can be argued that attending continuing education matches the concept of traditional resources. However, the item *participation in family conferences* does not seem to be consistent with the content of other items *(interactions with research nurses*, *quality improvement representatives and someone who champions research*) on the same factor, representing informal interactions of non-direct care providers (factor 8). Considering the short stay of patients in the ED, nurses in our sample may have found it difficult to relate to questions about patient rounds and family conferences as these may not be part of formal interactions in EDs. In fact, formal interactions such as planned meetings or family conferences may be very limited in EDs and an ACT scale for ED nurses may benefit from fewer items on this concept.

Cronbach’s alpha for the formal interaction concept is also low and all four items correlate below the quality standard of > 0.3 with the rest of the scale. Problematic reliability measures for the concept of formal interactions has been a consistent finding in previous validation studies of the ACT [14, 15, 37, 43]. The tool developers ascribe this to the fact that items for this concept were not developed as a true scale but rather represent a list of items assessing the frequency of staff participating in meetings. Consequently, these items may not necessarily function as one theoretical concept and load onto other factors with a better conceptual match. Items relating to formal interactions were therefore not removed from the tool although a factor analysis has shown that items are not related [15, 37].

The concept of informal interactions includes the frequency of interactions with other health professionals. Our factor analysis aligns with the original study for two sub-types of informal interactions, namely interactions with non-direct care providers (*research nurses*, *quality improvement specialists and research champions*) and direct care-providers (*nurses*, *physicians*, *other healthcare providers and clinical specialists*) [14]. Different to the original study [14], items in our factor analysis loaded onto a third factor and included interactions with unspecific personnel in the form of *hallway talks* or *bedside teaching*. Our analysis suggests a third sub-type of informal interactions of ED nurses, namely a theme related to where the interaction takes place but not with whom. The item *occurrence of hallway talks* had a low item-scale correlation (0.23), suggesting that it does not fit well within the overall scale of informal interactions. As mentioned above, this can be explained by the nature of this concept that includes items potentially not associated with each other to represent one common concept [37]. It could also highlight that ED nurses had difficulty rating the frequency of hallway talks because common ED layouts do not comprise hallways as they would be found on standard hospital wards or in nursing homes, where the ACT was originally tested.

Resources is another key concept for context already defined in the PARIHS framework and our factor analysis showed clearly defined sub-types of structural and electronic resources in line with the original validation of the ACT. Estabrooks et al. 2009 [14] determined three sub-types of structural resources: formal resources such as *noticeboards*, *policies and clinical practice guidelines*; traditional resources such as *journals*, *textbook and library use*; and electronic resources such as *computerised decisions support systems*, *email reminders and websites*. It is unclear from the Estabrooks publication onto which factor the item *workshops/courses* loaded, although this item loads onto the sub-type of electronic resources in our study. Items of formal resources, electronic resources and traditional resources loaded onto the same factors as the original study. Additional items from informal interactions (*occurrence of informal bedside teaching*) and formal interactions (*attending continued education outside the hospital*) load onto the traditional resources factor, which differs from the original study. However, these items can be understood as traditional resources of learning and thus fit the “theme” of the factor comprising traditional resources. Keeping in mind that the concept of structural and electronic resources has three sub-concepts, each including potentially independent items, their factor loadings suggest some relations between the items on the three sub-types, supporting the tool developer’s choice of categorising items into sub-types.

Some of the measurement problems highlighted with the exploratory factor analysis resonate in the results of the confirmatory factor analysis. The development of the three concepts informal interactions, formal interactions and structural/electronic resources as lists with independent items within each concept rather than scales with conceptually related items, is reflected in the model fit statistics. The purpose of a CFA is to assess a pre-defined categorisation of conceptually similar items and applying this to the three count-based concepts that comprised non-related items, we expected limited model fit ([15, 37]). This is consistent with the results of previous research in Canada [15, 37] and Germany [42] testing three separate models with i) all 10 concepts, ii) seven concepts including the true scale concepts and iii) three concepts including the count-based concepts. Similar to our CFA, models with the three count-based concepts performed consistently lower than the other 2 models [15, 37, 42]. Across all model-fit statistics the second model comprising true scale concepts performed slightly better than the model including all concepts. Accepting that this is due to the inclusion of the three count-based concepts, which the tool developers purposefully added to the ACT, our study supports the use of the ACT in an ED nurse population. However, the results also show that the ACT may benefit from amendments to individual items and concepts that better reflect the ED environment.

This study has some limitations. While the ACT is available for other health professions besides nurses, we collected perceptions on organisational context from ED nurses only. Since our trial focused on nurse-initiated care, we believe nurses were the most appropriate professional group to assess their work environment for this study. Therefore, validation of the ACT in an Australian ED nurse population refers to the ACT nurse survey only.

We acknowledge that the ACT is a self-report tool and thus responder bias may have been introduced. In a separate analysis (to be published elsewhere) we tested the association of responder and ED characteristics with the rating of context but could not identify significant influences on how individuals rated their work context.

While we used stratified random sampling, nurse unit managers self-selected eligible staff to complete the survey. For practical reasons it was not possible to apply a truly random selection strategy for this study.

The study comprised EDs of four Australian states and territories. In Australia, hospitals are managed at state-level so that EDs with various organisational systems were included in the study. This increases our confidence that findings are applicable to other EDs nationally and potentially internationally.

## Conclusions

Findings of our validation study in Australian ED settings provide evidence for the feasibility, validity and reliability of the ACT. While some conceptual shortcomings for individual concepts were detected, these can generally be explained by the tool developers’ choice of concept and item construction. Other limitations may be related to the application of the ACT in an ED environment. The ACT was developed based on data from nurses in nursing homes and non-acute wards and some items to assess context do not relate well to the specific high-acuity work context of EDs. While our validity and reliability analysis predominantly showed good results for the ACT in an ED nurse population, an amended ACT version for the ED environment may yield even better results.

In lieu of another tool to specifically capture ED context and its potential impact on clinicians’ use of research evidence, the ACT can be used by clinicians and researchers who are involved in implementation projects. A strength of the ACT is that high concept scores are indicative of higher use of research in clinical practice. Individual concepts of the ACT can provide feedback about leadership qualities and availability of resources and may be an important tool for clinical staff to highlight and negotiate their conditions of work.

## Supporting information

S1 Appendix Factor analysis item loadings (rotation matrix). (DOC)
