# Supplementary material for: Validating the Alberta Context Tool in a multi-site Australian Emergency Department nurse population
Source: PLoS One. 2019 Apr 9;14(4):e0215153. doi: 10.1371/journal.pone.0215153 (PMC6456203; doi:10.1371/journal.pone.0215153)
Supplement: S1 Appendix — (DOC) [file pone.0215153.s001.doc]

S1 Appendix

Factor analysis item loadings (rotation matrix)

| ACT Concept Items | Factor | | | | | | | | | | | | | |
| --- | --- | --- | --- | --- | --- | --- | --- | --- | --- | --- | --- | --- | --- | --- |
|  | 1 | 2 | 3 | 4 | 5 | 6 | 7 | 8 | 9 | 10 | 11 | 12 | 13 | 14 |
| Feedback 1 - receive feedback about team performance | 0.74 |  |  |  |  |  |  |  |  |  |  |  |  |  |
| Feedback 2 - team routinely discusses feedback | 0.74 |  |  |  |  |  |  |  |  |  |  |  |  |  |
| Feedback 3 - team has formal process for discussions | 0.76 |  |  |  |  |  |  |  |  |  |  |  |  |  |
| Feedback 4 - team routinely formulates action plans | 0.81 |  |  |  |  |  |  |  |  |  |  |  |  |  |
| Feedback 5 - team routinely monitors performance | 0.79 |  |  |  |  |  |  |  |  |  |  |  |  |  |
| Feedback 6 - team routinely compares performance | 0.75 |  |  |  |  |  |  |  |  |  |  |  |  |  |
| Leadership 1 - leader looks for feedback |  | 0.67 |  |  |  |  |  |  |  |  |  |  |  |  |
| Leadership 2 - leader focuses on success |  | 0.64 |  |  |  |  |  |  |  |  |  |  |  |  |
| Leadership 3 - leader handles stressful situations |  | 0.79 |  |  |  |  |  |  |  |  |  |  |  |  |
| Leadership 4 - leaders actively listens |  | 0.83 |  |  |  |  |  |  |  |  |  |  |  |  |
| Leadership 5 - leaders actively mentors |  | 0.74 |  |  |  |  |  |  |  |  |  |  |  |  |
| Leadership 6 - leader resolves confilcts |  | 0.75 |  |  |  |  |  |  |  |  |  |  |  |  |
| Connections 1 - the team shares information with each other |  |  | 0.55 |  |  |  |  |  |  |  |  |  |  |  |
| Connections 2 - my patient observations are taken seriously by superiors |  |  | 0.70 |  |  |  |  |  |  |  |  |  |  |  |
| Connections 3 - other teams share information with my team |  |  | 0.66 |  |  |  |  |  |  |  |  |  |  |  |
| Connections 4 - I am comfortable discussing patient care with superiors |  |  | 0.68 |  |  |  |  |  |  |  |  |  |  |  |
| Connections 5 - the aim of team work is to help others do their job |  |  | 0.66 |  |  |  |  |  |  |  |  |  |  |  |
| Connections 6 - those participating in group activities are valued by the team |  |  | 0.70 |  |  |  |  |  |  |  |  |  |  |  |
| Culture 1 - recognition from others |  |  |  | 0.62 |  |  |  |  |  |  |  |  |  |  |
| Culture 2 - control over my work |  |  |  | 0.65 |  |  |  |  |  |  |  |  |  |  |
| Culture 3 - balance of best practive and productivity |  |  |  | 0.64 |  |  |  |  |  |  |  |  |  |  |
| Culture 4 - undertake professional development |  |  |  | 0.55 |  |  |  |  |  |  |  |  |  |  |
| Culture 5 - work to provide what patients need |  |  |  | 0.56 |  |  |  |  |  |  |  |  |  |  |
| Culture 6 - member of a supportive group |  | 0.36 |  | 0.53 |  |  |  |  |  |  |  |  |  |  |
| Resources 1 - use of library |  |  |  |  | 0.63 |  |  |  |  |  |  |  |  |  |
| Resources 2 - use of text books |  |  |  |  | 0.74 |  |  |  |  |  |  |  |  |  |
| Resources 3 - use of journals |  |  |  |  | 0.59 |  |  |  |  |  |  |  |  |  |
| Resources 4 - use of notice boards |  |  |  |  |  |  |  |  |  | 0.38 |  |  |  |  |
| Resources 5 - use of policies |  |  |  |  |  |  |  |  |  | 0.85 |  |  |  |  |
| Resources 6 - use of clinical practice guideliens |  |  |  |  |  |  |  |  |  | 0.84 |  |  |  |  |
| Resources 7 - use of workshops in the hospital |  |  |  |  |  |  |  |  | 0.51 |  |  |  |  |  |
| Resources 8 - use of computerised decision support |  |  |  |  |  |  |  |  | 0.57 |  |  |  |  |  |
| Resources 9 - use of reminder systems |  |  |  |  |  |  |  |  | 0.71 |  |  |  |  |  |
| Resources 10 - use of websites |  |  |  |  |  |  |  |  | 0.68 |  |  |  |  |  |
| Informal interactions 1 - with someone in your profession |  |  |  |  |  | 0.80 |  |  |  |  |  |  |  |  |
| Informal interactions 2 - with physician |  |  |  |  |  | 0.75 |  |  |  |  |  |  |  |  |
| Informal interactions 3 - with other professional than phys or nurse |  |  |  |  |  | 0.71 |  |  |  |  |  |  |  |  |
| Informal interactions 4 - with research nurse |  |  |  |  |  |  |  | 0.63 |  |  |  |  |  |  |
| Informal interactions 5 - with clinical educator/specialist |  |  |  |  |  | 0.37 |  |  |  |  |  |  |  |  |
| Informal interactions 6 - with quality improvement representative |  |  |  |  |  |  |  | 0.66 |  |  |  |  |  |  |
| Informal interactions 7 - with someone who champions research |  |  |  |  |  |  |  | 0.71 |  |  |  |  |  |  |
| Informal interactions 8 - hallway talk |  |  |  |  |  |  |  |  |  |  |  |  |  | 0.78 |
| Informal interactions 9 - bedside teaching session |  |  |  |  | 0.39 |  |  |  |  |  |  |  |  | 0.38 |
| Time 1 - to do something extra for patients |  |  |  |  |  |  | 0.66 |  |  |  |  |  |  |  |
| Time 2 - to talk to someone about patient care plan |  |  |  |  |  |  | 0.72 |  |  |  |  |  |  |  |
| Time 3 - to look something up (e.g. journal/website) |  |  |  |  |  |  | 0.70 |  |  |  |  |  |  |  |
| Time 4 - to talk to someone about new clinical knowledge |  |  |  |  |  |  | 0.54 |  |  |  |  |  |  |  |
| Formal interactions 1 - team meetings |  |  |  |  |  |  |  |  |  |  |  |  |  |  |
| Formal interactions 2 - patient rounds |  |  |  |  |  |  |  |  |  |  |  |  | 0.79 |  |
| Formal interactions 3 - family conferences |  |  |  |  |  |  |  | 0.46 |  |  |  |  |  |  |
| Formal interactions 4 - continuing education outside hospital |  |  |  |  | 0.53 |  |  |  |  |  |  |  |  |  |
| Staffing 1 - enough staff to get necessary work done |  |  |  |  |  |  |  |  |  |  | 0.79 |  |  |  |
| Staffing 2 - enough staff to deliver quality care |  |  |  |  |  |  |  |  |  |  | 0.77 |  |  |  |
| Space 1 - adequate space to provide patient care |  |  |  |  |  |  |  |  |  |  | 0.51 | 0.38 |  |  |
| Space 2 - availability of private space to discuss confidential info |  |  |  |  |  |  |  |  |  |  |  | 0.90 |  |  |
| Space 3 - use of private space |  |  |  |  |  |  |  |  |  |  |  | 0.88 |  |  |
| Blue fields indicate items that loaded on the same factors as in the original validation study (Estabrooks et al. 2009); loadings > 0.35 are shown  Rotated factors are numbered by % of variance explained/sum of squared loadings  Rotated factors are numbered by % of variance explained/sum of squared loadings | | | | | | | | | | | | | | |
